# Travel to farms in the lowlands and inadequate malaria information significantly predict malaria in villages around Lake Tana, northwest Ethiopia: a matched case–control study

**DOI:** 10.1186/s12936-018-2434-y

**Published:** 2018-08-10

**Authors:** Asmamaw Malede, Kassahun Alemu, Mulugeta Aemero, Sirak Robele, Helmut Kloos

**Affiliations:** 1 Ethiopian Institute of Water Resources, Addis Ababa University, Addis Ababa, Ethiopia; 2 Department of Epidemiology and Biostatistics, Institute of Public Health, University of Gondar, Gondar, Ethiopia; 3 Department of Medical Parasitology, School of Biomedical & Laboratory Sciences, University of Gondar, Gondar, Ethiopia; 4 Department of Epidemiology and Biostatistics, University of California, San Francisco, USA

**Keywords:** Locally acquired malaria, Malaria information, Travel, Matched case–control study

## Abstract

**Background:**

In Ethiopia, malaria has declined in the last decade; only a small number of cases have been reported, primarily from hotspots. The contribution of house proximity to water bodies and the role of migration in malaria transmission has not yet been examined in detail in northwest Ethiopia. Individual and household-level environmental and socio-demographic drivers of malaria heterogeneity were explored contextually in meso-endemic villages around Lake Tana, northwest Ethiopia.

**Methods:**

A health facility-based paired age-sex matched case–control study involving 303 matched pairs was undertaken from 10 October 2016, to 30 June 2017. Geo-referencing of case households, control households, proximate water bodies, and health centres was carried out. A pretested and structured questionnaire was used to collect data on socio-demography, household assets, housing, travel history, and malaria intervention measures. Medians (interquartile range) were computed for continuous variables. Pearson’s Chi square/Fisher’s exact test was used to detect significant differences in proportions. Principal component analysis was performed to estimate household wealth. Stratified analysis was used to confirm confounding and interaction. A multivariable conditional logistic regression model was used to detect risk factors for malaria.

**Results:**

Of 303 malaria cases, 59 (19.5% [15.4–24.3]) were imported malaria cases whereas 244 (80.5% [75.7–84.6]) were locally acquired malaria cases. In bivariate analysis, marital status, educational status, and bed net ownership were significantly associated with malaria cases. In multivariable adjustment, travel to malarious lowlands in the preceding month (adjusted mOR = 7.32; 95% CI 2.40–22.34), household member’s travel to malarious lowlands (adjusted mOR = 2.75; 95% CI 1.02–7.44), and inadequate health information on malaria (adjusted mOR = 1.57; 95% CI 1.03–2.41) were predictors of malaria. Stratified analysis confirmed that elevation of households and travel to malarious lowlands were not effect modifiers. Travel to malarious lowlands had a confounding effect on malaria but elevation of households did not.

**Conclusions:**

In this study, travel to farms in the lowlands and inadequate health information on malaria were risk factors for malaria in villages around Lake Tana. This evidence is critical for the design of improved strategic interventions that consider imported malaria cases and approaches for accessing health information on malaria control in northwest Ethiopia.

**Electronic supplementary material:**

The online version of this article (10.1186/s12936-018-2434-y) contains supplementary material, which is available to authorized users.

## Background

Malaria still threatens the lives of millions of people, predominantly in sub-Saharan Africa; incidence increased between 2014 and 2016 in the WHO African region [1]. WHO targeted to eliminate malaria from at least 35 countries in which malaria was transmitted in 2015 and prevent re-establishment of malaria in all countries that are malaria free by 2030 through locally tailored interventions [2]. Wide use of effective interventions, including long-lasting insecticidal nets (LLINs), indoor residual spraying (IRS), treatment based on microscopy/rapid diagnostic tests (RDTs), and artemisinin-based combination therapy (ACT) has led to a considerable reduction in global malaria burden [3]. However, delivering interventions that deny the exceptional role of hot spots will extend the time needed for malaria elimination [4].

Over three-quarters of the Ethiopian landmass is malarious and 68% of the population is at risk for contracting malaria, depending largely on altitude and season [5]. This signifies a critical public health concern in a severely resource-limited country. Malaria has declined in the last decade, with a small number of cases reported, particularly from hotspots [1]. A 2015 malaria indicator survey using microscopy reported 0.4% malaria prevalence among people of all ages who travelled away from their home in the month preceding the survey and 0.5% among those who did not travel [5]. To end malaria, Ethiopia has employed three crucial interventions: distribution of bed nets, IRS in epidemic-prone areas, and enhanced diagnostic testing through RDT and microscopy along with deployment of ACT. As a result of intensive and integrated intervention measures, malaria incidence declined by 50–75% between 2000 and 2015 in the country [6]. This included implementing a program to eliminate locally acquired malaria transmission in 50 districts by 2020. Despite the gains made to date, sizeable residual transmission remains. This localized transmission is determined by demographic, socioeconomic, and environmental factors [7].

Malaria heterogeneity is multifactorial [7–9]. For instance, different environmental exposures increase malaria heterogeneity, particularly in rural settings; these include land cover, altitude, topography, human and vector population densities, and construction of residential houses [10]. Previous studies showed socio-demography [7, 11], household (HH) wealth [12], housing structure [13], travel history [7, 14] and intervention measures [8, 15] to be major factors in malaria transmission. Agricultural labourers often travel to malarious lowlands to work on commercial farms [7]. The resultant acquisition of locally acquired and imported malaria in meso-endemic areas hinders effective interventions in midland and highland areas bordering hyper-endemic areas [16, 17]. In addition, house proximity to water bodies has an inconsistent effect on malaria transmission. Some studies concluded that closeness to water bodies increases malaria risk [18, 19] while others reported lower risk near surface water [20, 21]. Some studies asserted the independence of malaria risk and house proximity to water bodies [22].

The average flight range of the main vector, *Anopheles gambiae*, is reported to be approximately 500 m [23]. Over the last 15 years, malaria prevalence has been decreasing in most endemic settings, due to the aforementioned integrated control intervention strategy, and in some settings the disease has been confined to hot spots with specific vector ecology conditions [4]. For malaria elimination and eradication, vector ecology is important. However, there are several ecological obstacles to vector control with existing interventions, including variations in mosquito behaviour, insecticide resistance, avoidance behaviour, vector biodiversity, competitive and food web interactions, dispersal and mating behaviour, and environmental change [24].

Comprehending heterogeneity in malaria exposure produces opportunities for spatially oriented malaria control [25]. Targeted malaria interventions have direct benefits both for targeted populations and for the whole community. Targeted control efforts are vital tools in malaria elimination when malaria transmission is sustained in hot spots [1].

Malaria elimination may be facilitated by finer-scale spatial studies. In areas with low malaria incidence, passive case detection (PCD) may lead to the identification of households (HHs) with more infections than others that can be a source of new infections. Determining the distance between HHs and the proximate water bodies where symptomatic malaria cases originate may help to define hotspots of malaria and contribute to improving malaria elimination efforts by optimizing the delivery of limited resources to higher-risk populations [26, 27]. The role of GPS-based house proximity to water bodies on malaria has not yet been documented in northwest Ethiopia, and no studies have traced the potential role of imported malaria in maintaining transmission foci in the country. This study aimed to determine the effect of house proximity to water bodies and other underlying factors on malaria heterogeneity in villages around Lake Tana. Specifically, this study explored the role of socio-demographic characteristics, travel history, HH wealth, housing structure, and malaria intervention measures on malaria heterogeneity. The findings of this study may guide national malaria control and elimination programmes in re-designing programmes for malaria elimination in Ethiopia.

## Methods

### Study area and settings

**Fig. 1:**
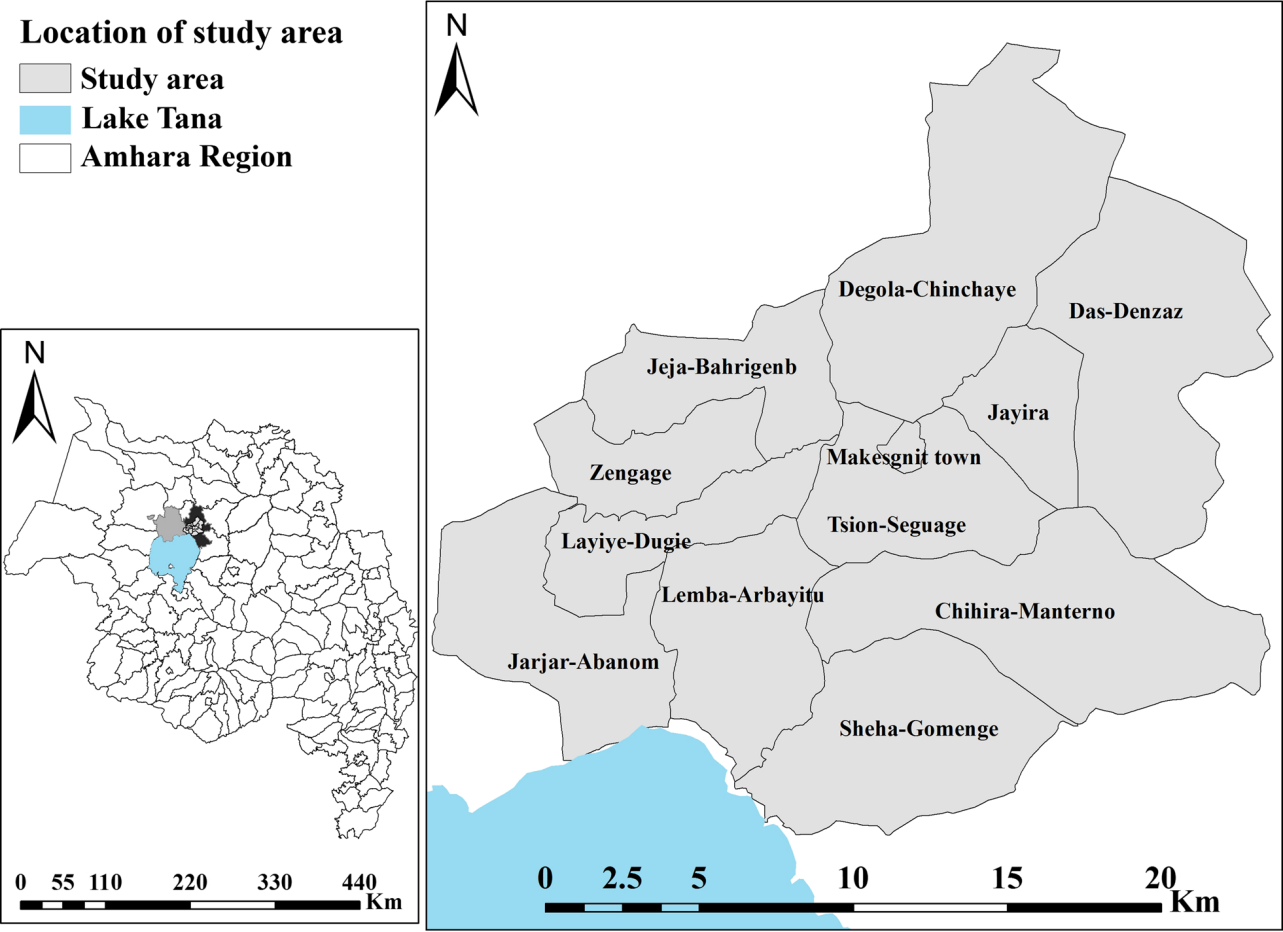
Geographic location of study area around Lake Tana, northwest Ethiopia

The study was undertaken in 11 *kebeles* (the lowest administrative units in Ethiopia) of Gondar *Zuriya* District and 1 *kebele* of *Dembia* District, northwest Ethiopia, from 10 October 2016, to 30 June 2017 (Fig. 1). Out of 37 *kebeles* in Gondar *Zuriya* District, eight are not endemic for malaria. In 2016/2017, Gondar *Zuriya* and *Dembia* districts had a projected population of 222, 700 and 315, 390, respectively. The area of Gondar *Zuriya* District is 1108.53 km^2^, with a population density of 188.4 persons/km^2^ [28]. Gondar Zuriya District is composed of hilly and plain landscapes, and the altitude ranges from 1750 to 2600 m above sea level over three highland fringe zones. Subsistence agriculture is the main occupation of the inhabitants. In 2016, total rainfall, mean maximum temperature, mean minimum temperature and relative humidity in the study area were 1047.6 mm, 27.4 °C, 14.7 °C, and 45%, respectively [29]. Gondar Zuriya District has seven health centres and 38 health posts that provide health services for the people in the district.

Since 2004, guidelines on malaria case management in the country have recommended treatment based on parasitological diagnosis with microscopy/RDTs. Artemether–lumefantrine is the first-line drug in Ethiopia. Seasonally, large numbers of malaria cases are reported from September to December in northwest Ethiopia, mostly *Plasmodium falciparum* infections [30]. In Gondar *Zuriya* District, large number of individuals travel to malarious lowlands to temporarily work on commercial agricultural farms, mainly during the rainy and spring seasons. Males aged 15 years and above are the main travellers to the lowlands. Malaria is a common seasonal disease in the district. A 10-year (2002–2011) trend analysis conducted in a neighboring district’s health centre reported 23,473 microscopically confirmed malaria cases (75% *P. falciparum* and 25% *Plasmodium vivax*) with a fluctuating trend and mean malaria cases of 2, 347. From 2010 to 2011, *P. falciparum* prevalence decreased while *P. vivax* prevalence was increasing, indicating a trend shift from *P. falciparum* to *P. vivax*. Males and those in the age group of 15–44 years were highly affected [30]. Another study conducted at selected health institutions in three districts reported 61.3% prevalence of malaria (72.3% *P. falciparum* and 27.7% *P. vivax*) microscopically [31]. There is an annual IRS programme and distribution of LLINs, with replacement being done every 3 years. Bendiocarb and Propoxur has been used for spraying interior walls of houses and LLINs (DuraNet^®^) have been distributed since September 2005. One study recognized *Anopheles arabiensis* as a primary vector of malaria around Lake Tana [32].

The study area has altitudes of 1750–2500 m above sea level [33]. The climate of Lake Tana basin is of the temperate tropical highland type [34]. Most rain falls between June and September [34]. The water of Lake Tana is turbid and the lake’s shores are becoming covered with water hyacinth. Gumara Stream flows from east to west and into Lake Tana, irrigating extensive commercial garlic farms along its course. The irrigation water is pumped using generators, causing a substantial decrease in river flow during the dry season (October to February). The reduction in river flow creates stagnant areas, potential breeding habitats for mosquitoes.

### Study design and eligibility criteria

A health facility-based paired-matched case–control study was used. Cases and controls were individually matched by sex. However, frequency matching was done by age (< 15, 15–49, and > 49 years). All patients ≥ 5 months of age who visited *Lemba* and *Makesegnit* health centres and permanent residents in the study *kebeles* were included. Individuals who had no *Plasmodium*-positive blood film or symptoms of malaria during the preceding month confirmed using patient interview or health centre register were enrolled in the control group. Individuals with new episodes of malaria or people with recrudescent cases that presented after 1 month or more of the initial infection, and those who had taken malaria treatment in the previous 30 days were excluded from the study.

### Study population

The patients (malaria cases and controls) were included into the study based on the clinical signs and symptoms of malaria and blood slides results. When patients came to the health centres for treatment, clinical diagnosis was performed for malaria. Then, the presence of *Plasmodium* parasites in the blood samples was confirmed using malaria microscopy. When a malaria case was found, the matched control was obtained from the patients by confirming through clinical diagnosis and slide results within 3 days after the case was diagnosed.

### Sample size computation

Sample size was determined using matched case–control study design sample size calculation methods [35] with an assumption of 95% level of confidence, 80% power to detect exposure difference, odds ratio (OR) of 1.61, one control per case, and assuming that 25% of the control HHs were located within a 250 m radius of water bodies [26]. Based on these assumptions, 333 case–control matched pairs could be included in the study.

### Data collection and quality assurance

Microscopy was used to confirm the presence of *Plasmodium* species in the clinically diagnosed patients [36]. Microscopy is the gold standard method of diagnosing malaria [37]. Confirmation of positivity or negativity of slide results was assured using standard operating procedures of Giemsa malaria microscopy at the health centres. Thick blood film was used to search for malaria parasites while thin film was used to confirm the malaria parasite species when this could not be done in the thick film. Malaria diagnoses were performed at the two health centres and patients were selected and classified as cases or controls based on the eligibility criteria. Cases were patients who had signs and symptoms of malaria or reported fever during the previous 48 h and were positive for single or mixed *Plasmodium* species in a blood sample diagnosed by microscopy [36]. Controls were individuals presenting at the health centres whose thick blood smear tests were negative for malaria. Experienced microscopists who were certified by the Ethiopian Federal Ministry of Health double checked all the slides at the health centres.

All tested slides (negative and positive) were stored at the health centres and three quarters of the sample were collected randomly for quality control. Blind examination of the slides was independently performed by a senior technician at the University of Gondar Specialized Hospital Laboratory.

A pre-tested, semi-structured questionnaire was used to collect individual and HH information. The questionnaire consisted of questions on socio-demography, HH assets, housing structure, LLINs, IRS, travel history, visits to traditional healers, and inappropriate home-based management of malaria (HMM). Males who completed grade 12 collected the individual and HH data through HH visits. Data collectors were trained in field data collection for 3 days by the lead researcher. If any case or control was absent at the first visit, the HH was revisited within 2 days. After the second visit, 15 HHs were excluded and 15 subjects refused to be interviewed.

Geo-referencing of case HHs, control HHs, small water bodies, a part of the shore of Lake Tana, and the two health centres was recorded through a handheld GPS device (Garmin’s GPSMAP 60CSx, Garmin International Inc., USA) with an accuracy goal of ~ 5 m. Geo-locations were also recorded in notebooks and checklists. An additional image file shows the characteristics of study villages in more detail (see Additional file 1). Field data collectors were blinded to the malaria status of the enrolled individuals. The questionnaire was prepared in English, translated into the Amharic language, and the responses from the study participants were translated to English by independent language experts.

### Variables and measurements

**Fig. 2:**
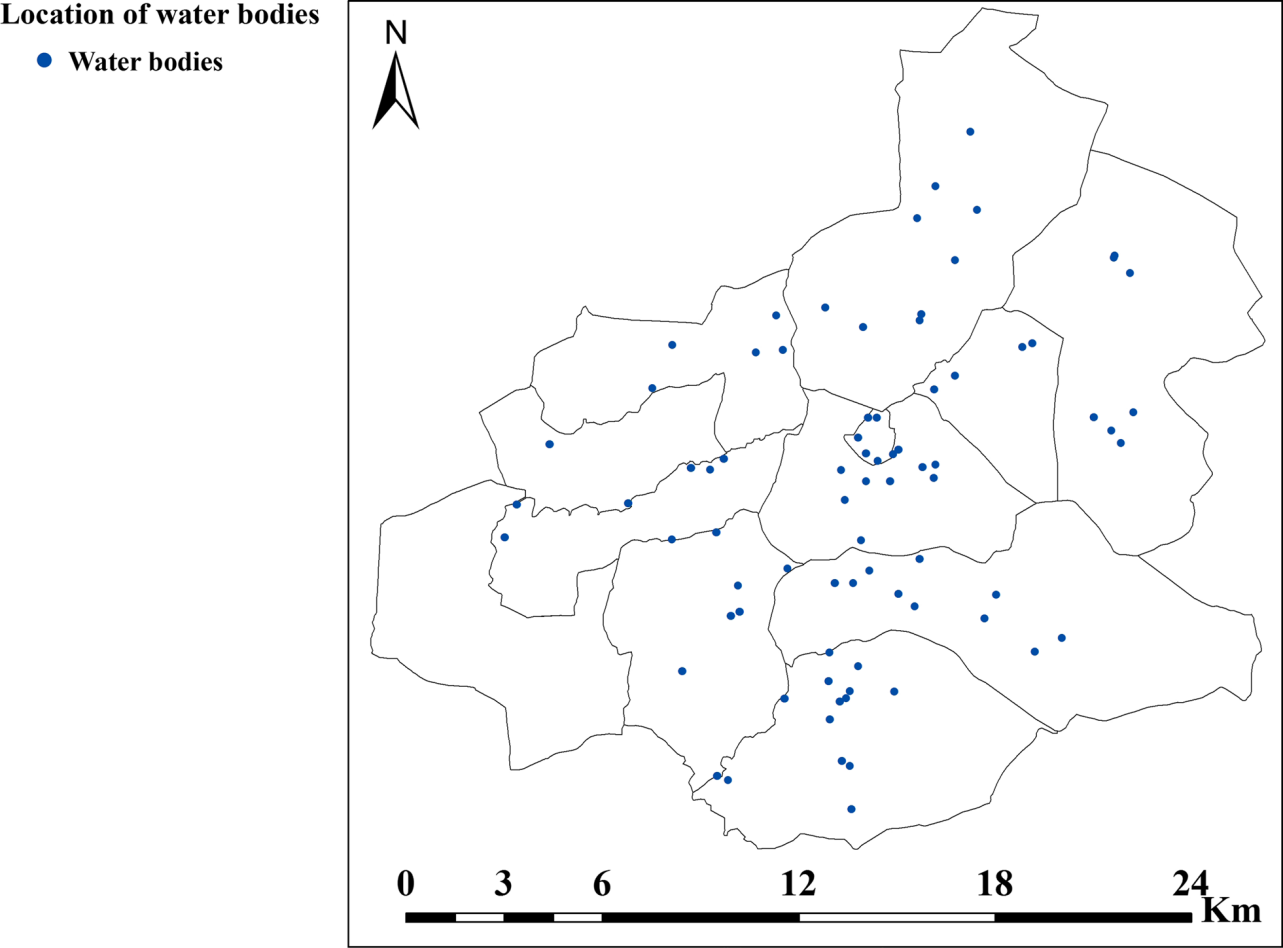
Location of proximate water bodies to case HHs and control HHs

House proximity to water bodies within a 250 m radius was the primary exposure variable. The remaining variables were covariates. The outcome variable was malaria status (dichotomous). Through an ecological survey, two nearby water bodies (rivers, streams, marshes, swamps, stagnant water, natural pools, springs, water holes) were georeferenced for each case HH and control HH (Fig. 2). Then, by observational estimation, water bodies within a 500 m radius of HHs were grouped in Category 1 (water body 1) and those farther away from the HHs were grouped in Category 2 (water body 2).

For analysis, house proximity to water bodies and health centres was computed as Euclidean distance using GPS coordinates [38, 39]. The average flight range of the main vector *An. gambiae* is reported to be approximately 500 m [23]. For this study, house proximity to water bodies was categorized into three distance bands (< 250 m, 250–500 m and > 500 m) [40]. The Euclidean distance of each HH to the health centre was categorized as a dichotomous explanatory variable (≤ 5 km and > 5 km) [41].

HH wealth reflects a composite of several dichotomous and polychotomous variables used to indicate the socioeconomic status of the HH. Variables that used for HH wealth index construction were developed on the basis of literature [42, 43] and the Ethiopian Demographic and Health Survey [44]. Longer-run HH assets, housing structure, water supply source, sanitation facility, agricultural land size, having domestic animals, and having a savings account were used to construct the wealth index. The values of the variables were weighted from 0 to 1, indicating the lowest and the highest values, respectively.

### Statistical analysis

Data were double entered, coded, cleaned, and verified using *EpiData Version 3.1* software (EpiData Association, Odense, Denmark). Data analysis was carried out using SPSS statistical software version 22 (IBM Corp. IBM SPSS Statistics for Windows, Armonk, NY, USA) and STATA version 14.0 (StataCorp. Statistical Software: College Station, TX 77845, USA). Descriptive statistics [*n* (%)], including median IQR (interquartile range), were calculated for continuous variables. For categorical variables, Chi squared or Fisher’s exact test were employed to detect significant differences in proportions. All reported *p*-values were two-tailed and considered statistically significant if *p *< 0.05.

Principal component analysis (PCA) was used to estimate HH wealth [42]. Principal component 1 was used to rank the HHs into three groups. Cutoff values/rank categories were set for the three groups. The original variables were transformed into scores having a mean equal to zero and standard deviation equal to one (standardization). The value of each standardized variable was multiplied by principal component 1 and a third variable created from the product for each variable. Finally, a HH wealth score was computed from the sum of the third variables. Based on the cutoff values of principal component 1 ranks, a final three-category HH wealth index variable was developed. The composite score was classified into tertiles, with the higher tertile reflecting higher wealth.

Cochran Mantel–Haenszel stratified analyses were employed to eliminate co-factors, and to discover and describe effect modifiers. Mantel–Haenszel test of equality of stratum-specific ORs was used to identify effect modifiers. First, the equality of stratum-specific ORs were checked to confirm significant differences, which indicate the presence of effect modification between the exposure and other factors. If not different, the presence of confounding was then assessed. The Mantel–Haenszel test produced a single weighed estimate of the predictor by adjusting the effects of the third variable and the crude estimate. Then, confounding presence was ascertained through degree of difference between the crude and adjusted ORs. For confirmation, a relative difference in the effect estimate with a cutoff of ≥ 10% was used for evidence of confounding [45].

The conditional logistic regression model is the standard tool for the analysis of matched case–control studies. It allows for the analysis of data with stratified samples. Like other regression models, it allows for multiple variables, continuous exposures, confounding, and effect-modifying variables to be handled appropriately [46]. Although age was a matching variable, it was included in the model to eliminate the residual confounding effect that may come from broad age categories. Through a matching variable for cases and controls, a matched analysis was employed to explore factors associated with malaria infection. The modelling strategy involved estimating unadjusted matched odds ratio (mOR) and adjusted mOR of the studied variables with malaria infection at 95% CI. A bivariate analysis was executed for each variable; those variables having p < 0.2 along with variables having biological plausibility were included in the multivariable model. β-coefficients, ORs with 95% confidence intervals (CI) and p-values were estimated for the multivariable matched analysis and variables at p < 0.05 were considered as statistically significant and independently associated with malaria infection.

## Results

### Socio-demographic and household physical characteristics of cases and controls

**Fig. 3:**
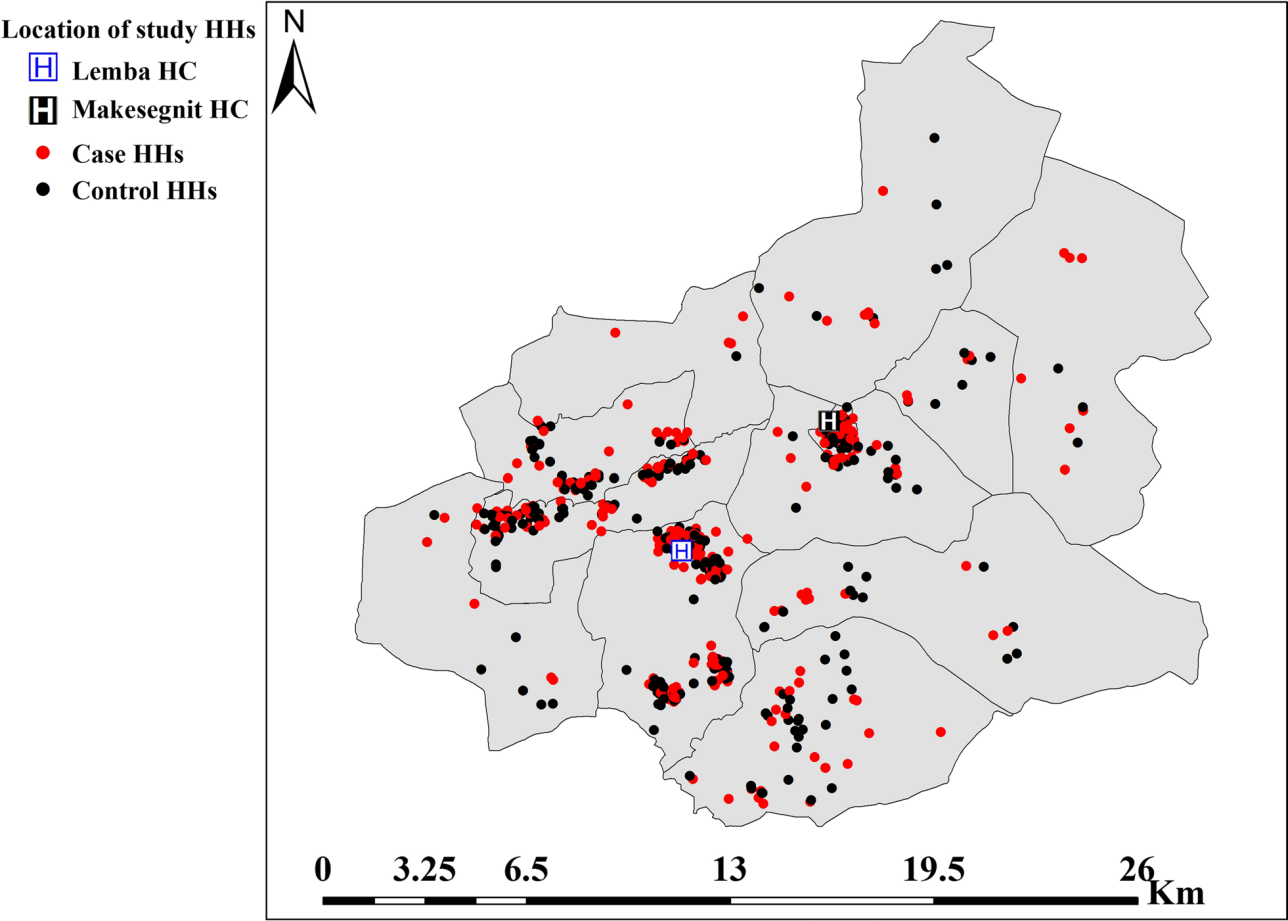
Location of case HHs, control HHs and health centers

A total of 606 patients (303 case–control matched pairs) participated in the study, and their HH geo-location was mapped (Fig. 3). Thirty matched pairs were ineligible for the study because they could not be contacted using the addresses they gave to the health centre during diagnosis, finding matched controls for cases within 3 days, and refusal to enroll, giving a response rate of 90.9%. Eight patients refused to participate in the study. The quality control results showed 98.5% inter-rater agreement and kappa coefficient of 0.97 with the results at the health centres (*p *< 0.0001). Discordant results were reread by a third senior technician and this result was taken as the final result.

The age distribution of cases and controls for matching was as follows: 238 (39.3% [35.5–43.2]) for subjects < 15 years of age, 328 (54.1% [50.1–58.1]), for subjects 15–49 years of age, and 40 (6.6% [4.9–8.9]), for subjects > 49 years of age. Half of the malaria infections, 152 (50.3% [44.5–55.8]), were *P. vivax* mono-infections while the other half (48.8%) were *P. falciparum* mono-infections, with only few mixed infections. Of the total malaria cases, 108 (35.6% [30.4–41.2]) occurred between September 15, 2016, and December 15, 2016; 71 (23.4% [19.0–28.6]) occurred between December 16, 2016, and March 15, 2017; and 124 (40.9% [35.5–46.6]), occurred between March 16, 2017, and June 30, 2017. The median (IQR) age of the malaria cases was 18 (4.42–30) years. Of the 303 malaria cases, 199 (65.7% [61.8–69.4]) were males (Table 1).

**Table 1:** Socio-demographic and housing characteristics of cases and controls in villages around Lake Tana, Ethiopia, October 2016–June 2017

Characteristic	Malaria cases (n = 303) n, % (95% CI)	Controls (n = 303) n, % (95% CI)	Total n, % (95% CI)	Pearson’s X^2^/Fisher’s exact test**
Age (years)^g^	18 (4.42–30)	20 (3–33)	19 (4–30)	0.188
< 15	119, 39.3 (33.9–44.9)	119, 39.3 (33.9–44.9)	238, 39.3 (35.5–43.2)	
15–29	108, 35.6 (30.4–41.2)	90, 29.7 (24.8–35.1)	198, 32.6 (29.0–36.5)	
30–44	42, 13.8 (10.4–18.3)	62, 20.5 (16.3–25.4)	104, 17.2 (14.4–20.4)	
45–64	30, 9.9 (7.0–13.8)	30, 9.9 (7.0–13.8)	60, 9.9 (7.8–12.6)	
≥ 65	4, 1.3 (0.5–3.5)	2, 0.6 (0.2–2.6)	6, 1.0 (0.4–2.2)	
Religion^a^				0.105
Orthodox Christian	299, 98.7 (96.5–99.5)	293, 96.7 (94.0–98.2)	592, 97.7 (96.1–98.6)	
Muslim	4, 1.3 (0.5–3.5)	10, 3.3 (1.8–6.0)	14, 2.3 (1.4–3.9)	
Marital status^a^				0.068
Married	235, 77.6 (72.5–81.9)	257, 84.8 (80.3–88.5)	492, 81.2 (77.9–84.1)	
Single	48, 15.8 (12.1–20.4)	34, 11.2 (8.1–15.3)	82, 13.5 (11.0–16.5)	
Divorced/widowed	20, 6.6 (4.3–10.0)	12, 4.0 (2.3–6.9)	32, 5.3 (3.8–7.4)	
Educational status^a^				0.175
Illiterate^f^	140, 46.2 (40.6–51.9)	162, 53.5 (47.8–59.0)	302, 49.8 (45.9–53.8)	
Able to read and write (informal)	63, 20.8 (16.6–25.8)	55, 18.2 (14.2–22.9)	118, 19.5 (16.5–22.8)	
Grade 1–12 completed	93, 30.7 (25.7–36.1)	75, 24.7 (20.2–29.9)	168, 27.7 (24.3–31.4)	
> 12	7, 2.3 (1.1–4.8)	11, 3.6 (2.0–6.5)	18, 3.0 (1.9–4.7)	
Occupation^a^				0.435
Government employee	6, 2.0 (0.9–4.4)	10, 3.3 (1.8–6.0)	16, 2.6 (1.6–4.3)	
Merchant/trader	10, 3.3 (1.8–6.0)	9, 3.0 (1.5–5.6)	19, 3.1 (2.0–4.9)	
Farmer/peasant	251, 82.8 (78.1–86.7)	258, 85.2 (80.7–88.7)	509, 84.0 (80.8–86.7)	
Daily labourer	7, 2.3 (1.1–4.8)	8, 2.6 (1.3–5.2)	15, 2.5 (1.5–4.1)	
Others^b^	29, 9.6 (6.7–13.5)	18, 5.9 (3.8–9.2)	47, 7.8 (5.9–10.2)	
HH wealth index				0.275
Poor	89, 29.4 (24.5–34.8)	106, 35.0 (29.8–40.6)	195, 32.2 (28.6–36.0)	
Medium	20, 6.6 (4.3–10.0)	15, 5.0 (3.0–8.1)	35, 5.8 (4.2–7.9)	
Rich	194, 64.0 (58.4–69.3)	182, 60.0 (54.4–65.5)	376, 62.0 (58.1–65.8)	
HH size^g^	6 (4–7)	6 (4–7)	6 (4–7)	0.568
≤ 5 individuals	133, 43.9 (38.4–49.6)	140, 46.2 (40.6–51.9)	273, 45.0 (41.1–49.0)	
> 5 individuals	170, 56.1 (50.4–61.6)	163, 53.8 (48.1–59.4)	333, 55.0 (51.0–58.9)	
Roofing materials				0.145
Corrugated iron	287, 94.7 (91.5–96.7)	278, 91.8 (88.1–94.4)	565, 93.2 (90.9–95.0)	
Thatch	16, 5.3 (3.3–8.5)	25, 8.2 (5.6–11.9)	41, 6.8 (5.0–9.1)	
Open eaves in the roof				0.546
Yes	37, 12.2 (8.9–16.4)	42, 13.9 (10.4–18.3)	79, 13.0 (10.6–15.9)	
No	266, 87.8 (83.6–91.0)	261, 86.1 (81.7–89.6)	527, 87.0 (84.0–89.4)	
Wall building materials				0.896
Cement	5, 1.6 (0.7–3.9)	3, 1.0 (0.3–3.0)	8, 1.3 (0.7–2.6)	
Mud	296, 97.7 (95.2–98.9)	298, 98.3 (96.1–99.3)	594, 98.0 (96.5–98.9)	
Others^c^	2, 0.7 (0.2–2.6)	2, 0.7 (0.2–2.6)	4, 0.7 (0.2–1.7)	
Flooring materials				0.262
Cement and ceramics	9, 3.0 (1.5–5.6)	4, 1.3 (0.5–3.5)	13, 2.2 (1.2–3.7)	
Dung plastered	294, 97.0 (94.4–98.5)	299, 98.7 (96.5–99.5)	593, 97.8 (96.3–98.8)	
Windows of the house				0.464
Corrugated iron	55, 18.2 (14.2–23.0)	52, 17.2 (13.3–21.9)	107, 17.7 (14.8–20.9)	
No windows	182, 60.1 (54.4–65.5)	197, 65.0 (59.4–70.2)	379, 62.5 (58.6–66.3)	
Open windows/open wall	58, 19.1 (15.1–24.0)	50, 16.5 (12.7–21.1)	108, 17.8 (15.0–21.1)	
Others^d^	8, 2.6 (1.3–5.2)	4, 1.3 (0.5–3.5)	12, 2.0 (1.1–3.5)	
Number of rooms in the house^g^	3 (2–3)	3 (2–3)	3 (2–3)	0.549
≤ 2 rooms	100, 33.0 (27.9–38.5)	107, 35.3 (30.1–40.9)	207, 34.2 (30.5–38.0)	
> 2 rooms	203, 67.0 (61.5–72.1)	196, 64.7 (59.1–69.9)	399, 65.8 (62.0–69.5)	
Tethering of cattle, goats and sheep^e^				0.701
Yes	73, 24.1 (19.6–29.3)	69, 22.8 (18.4–27.9)	142, 23.4 (20.2–27.0)	
No	230, 75.9 (70.7–80.4)	234, 77.2 (72.1–81.6)	464, 76.6 (73.0–79.8)	
Separate kitchen				0.614
Yes	116, 38.3 (32.9–43.9)	110, 36.3 (31.1–41.9)	226, 37.3 (33.5–41.2)	
No	187, 61.7 (56.1–67.1)	193, 63.7 (58.1–68.9)	380, 62.7 (58.8–66.5)	

*CI* confidence interval

** *p* < 0.05 was considered statistically significant

^a^For cases and controls < 18 years; religion, marital status, educational status and occupation of the household head was taken

^b^Student, job seeker, street dweller, weaver, local musician, sewer, carter

^c^Cement and sand-made bricks, ink painted, ordered stones

^d^Wooden, glass sealed

^e^Tethering indoors at night in the month prior to visiting the health facility

^f^Unable to read and write based on self-reporting

^g^Continuous variables; summarized through median and IQR

### Travel to commercial agricultural farms and other behaviours among cases and controls

Almost all the study patients, 603 (99.5% [98.5–99.8]), had been active at night (6 pm to 12 pm) outside the home in the preceding month. Seventy (11.6% [9.2–14.4]) took anti-malarial drugs presumptively. In addition, 83 (13.7% [11.2–16.7]) travelled to malarious lowlands (Metema, Shehedi, Abderafi, Abrahajira, Humera, Maikadera, Dansha, Belessa and Fogera) in the preceding month. From those who travelled to malarious lowlands, 55 (66.3% [55.2–75.8]) were engaged in agriculture. The main reason for travel, cited by 75 patients (90.4% [81.7–95.2]), was to seek temporary jobs. Most of the travellers, (82, 98.8% [91.6–99.8]), were males; 58 (69.9% [59.0–78.9]) were 15–29 years of age; 39 (66.1% [52.8–77.3]) had *falciparum* malaria; and 42 (50.6% [39.8–61.4]) lived at elevations between 1901 and 2100 m above sea level. Of the 303 malaria cases, 59 (19.5% [15.4–24.3]) had imported malaria whereas 244 (80.5% [75.7–84.6]) had locally acquired malaria (Table 2).

**Table 2:** Travel history and other behaviours among cases and controls in villages around Lake Tana, Ethiopia, October 2016–June 2017

Characteristic	Malaria cases (n = 303) n, % (95% CI)	Controls (n = 303) n, % (95% CI)	Total n, % (95% CI)	Pearson’s X^2^/Fisher’s exact test**
Travel to malarious lowlands^a^				*< 0.0001*
Yes	59, 19.5 (15.4–24.3)	24, 7.9 (5.4–11.6)	83, 13.7 (11.2–16.7)	
No	244, 80.5 (75.7–84.6)	279, 92.1 (88.4–94.6)	523, 86.3 (83.3–88.8)	
Frequency of travel to malarious lowlands (n = 83)				0.714
One time	53, 89.8 (78.7–95.5)	21, 87.5 (65.5–96.3)	74, 89.2 (80.2–94.3)	
Two or three times	6, 10.2 (4.5–21.3)	3, 12.5 (3.7–34.5)	9, 10.8 (5.7–19.8)	
Regular LLIN use during travels (n = 83)				1.000
Yes	3, 5.1 (1.6–15.1)	1, 4.2 (0.5–27.3)	4, 4.8 (1.8–12.4)	
No	56, 94.9 (84.9–98.4)	23, 95.8 (72.7–99.5)	79, 95.2 (87.6–98.2)	
Reason for travel (n = 83)				1.000
To work temporarily/job seeking	53, 89.8 (78.7–95.5)	22, 91.7 (69.8–98.1)	75, 90.4 (81.7–95.2)	
Others^b^	6, 10.2 (4.5–21.3)	2, 8.3 (1.9–30.2)	8, 9.6 (4.8–18.3)	
Travel history within the study area^a^				*0.037*
Yes	95, 31.4 (26.4–36.8)	72, 23.8 (19.3–28.9)	167, 27.6 (24.1–31.3)	
No	208, 68.6 (63.2–73.6)	231,76.2 (71.1–80.7)	439, 72.4 (68.7–75.9)	
Regular LLIN use during travels (n = 167)				0.398
Yes	9, 9.5 (4.9–17.4)	4, 5.6 (2.1–14.2)	13, 7.8 (4.5–13.0)	
No	86, 90.5 (82.6–95.1)	68, 94.4 (85.8–97.9)	154, 92.2 (87.0–95.5)	
HH member travel to malarious lowlands^a^				*0.001*
Yes	29, 9.6 (6.7–13.5)	9, 3.0 (1.5–5.6)	38, 6.3 (4.6–8.5)	
No	274, 90.4 (86.5–93.3)	294, 97.0 (94.4–98.5)	568, 93.7 (91.5–95.4)	
Reason for travel (n = 38)				0.574
To work temporarily/job seeking	26, 89.7 (70.9–96.9)	7, 77.8 (33.0–96.1)	33, 86.8 (71.1–94.6)	
Others^b^	3, 10.3 (3.1–29.1)	2, 22.2 (3.9–67.0)	5, 13.2 (5.4–28.9)	
Visiting traditional healers^c^				0.412
Yes	55, 18.2 (14.2–22.9)	63, 20.8 (16.6–25.8)	118, 19.5 (16.5–22.8)	
No	248, 81.8 (77.1–85.8)	240, 79.2 (74.2–83.4)	488, 80.5 (77.2–83.5)	

** *p *< 0.05 was considered statistically significant. The italics indicate statistically significant *p*-values

^a^In the month prior to visiting the health centre

^b^For holy water, spiritual festivity, spiritual education, commercial issues, visiting friends and relatives, labour (playing music in resource rich areas of low lands)

^c^Spiritual healing, herbal remedy

### Intervention measures of malaria among cases and controls

The median (IQR) distance from house to health centre of the patients was 3.6 (0.9–5.3) km. Fewer than one-third of the patients, 185 (30.5% [27.0–34.3]) owned at least one LLIN per HH. Of those patients who had LLINs, 123 (66.5% [59.3–73.0]) reported having slept under a LLIN the night prior to the interviews. Only 95 (51.4% [44.1–58.5]) of HHs had LLINs that were in good condition. Overall, 451 (74.4% [70.8–77.8]) reported that their houses had been sprayed with IRS in the preceding year (Table 3).

**Table 3:** Malaria intervention measures among cases and controls in villages around Lake Tana, Ethiopia, October 2016–June 2017

Characteristic	Malaria cases (n = 303) n, % (95% CI)	Controls (n = 303) n, % (95% CI)	Total n, % (95% CI)	Pearson’s X^2^/Fisher’s exact test**
House proximity to health centre (km)^b^	3.5 (0.7–4.9)	3.8 (1.2–5.5)	3.6 (0.9–5.3)	0.065
≤ 5	233, 76.9 (71.8–81.3)	213, 70.3 (64.9–75.2)	446, 73.6 (69.9–77.0)	
> 5	70, 23.1 (18.7–28.2)	90, 29.7 (24.8–35.1)	160, 26.4 (23.0–30.1)	
Received health information on malaria				*0.011*
Yes	123, 40.6 (35.2–46.3)	93, 30.7 (25.7–36.1)	216, 35.6 (31.9–39.6)	
No	180, 59.4 (53.7–64.8)	210, 69.3 (63.9–74.3)	390, 64.4 (60.4–68.1)	
LLIN ownership				0.186
None	203, 67.0 (61.5–72.1)	218, 71.9 (66.6–76.7)	421, 69.5 (65.7–73.0)	
1 LLIN/household	52, 17.2 (13.3–21.9)	56, 18.5 (14.5–23.3)	108, 17.8 (15.0–21.1)	
≥2 LLINs/household	48, 15.8 (12.1–20.4)	29, 9.6 (6.7–13.5)	77, 12.7 (10.3–15.6)	
Slept under LLIN regularly in last 1 month (n = 185)^#^				0.085
Yes	38, 38.0 (28.9–48.0)	43, 50.6 (39.9–61.2)	81, 43.8 (36.7–51.1)	
No	62, 62.0 (52.0–71.1)	42, 49.4 (38.8–60.1)	104, 56.2 (48.9–63.3)	
LLIN(s) had holes/tear(s) (n = 185)				0.603
Yes	39, 39.0 (29.8–49.0)	30, 35.3 (25.7–46.2)	69, 37.3 (30.6–44.6)	
No	61, 61.0 (51.0–70.2)	55, 64.7 (53.8–74.3)	116, 62.7 (55.4–69.4)	
Period since the HH obtained LLIN(s) (n = 185) (year)				0.723
< 1	11, 11.0 (6.1–18.9)	8, 9.4 (4.7–17.9)	19, 10.3 (6.6–15.6)	
≥ 1	89, 89.0 (81.1–93.9)	77, 90.6 (82.1–95.3)	166, 89.7 (84.4–93.4)	
Proper hanging of LLIN(s) (n = 185)				0.747
Yes	72, 72.0 (62.3–80.0)	63, 74.1 (63.6–82.5)	135, 73.0 (66.1–78.9)	
No	28, 28.0 (20.0–37.7)	22, 25.9 (17.5–36.4)	50, 27.0 (21.1–33.9)	
Is/are the LLIN(s) in good condition? (n = 185)^a^				0.488
Yes	49, 49.0 (39.2–58.9)	46, 54.1 (43.3–64.6)	95, 51.4 (44.1–58.5)	
No	51, 51.0 (41.1–60.8)	39, 45.9 (35.4–56.7)	90, 48.6 (41.5–55.9)	
IRS				0.926
Yes	225, 74.3 (69.0–78.9)	226, 74.6 (69.4–79.2)	451, 74.4 (70.8–77.8)	
No	78, 25.7 (21.1–31.0)	77, 25.4 (20.8–30.6)	155, 25.6 (22.3–29.2)	
Plastering/painting after IRS (n = 451)				0.351
Yes	27, 12.0 (8.3–17.0)	21, 9.3 (6.1–13.9)	48, 10.6 (8.1–13.9)	
No	198, 88.0 (83.0–91.7)	205, 90.7 (86.1–93.9)	403, 89.4 (86.1–91.9)	

**p < 0.05 was considered statistically significant. The italics indicate statistically significant *p*-values

^a^Measured by service years of LLIN (aged if > 3 years), presence of tears/holes and proper hanging

^b^Continuous variables; summarized through median and IQR

### Proximity of case HHs and control HHs to water bodies

Two hundred twenty-four (73.9% [68.7–78.6]) of the case HHs were located at an altitude between 1701 and 1900 m above sea level. Only 18 (5.9% [3.8–9.3]) of case HHs were located within 2 km of Lake Tana. Likewise, 18 (5.9% [3.8–9.3]) case HHs had a water body 1 within a 250 m radius (Table 4).

**Table 4:** Household elevation and proximity to water bodies in villages around Lake Tana, Ethiopia, October 2016–June 2017

Variables	Malaria cases (n = 303) n, % (95% CI)	Controls (n = 303) n, % (95% CI)	Total n, % (95% CI)	Pearson’s X^2^/Fisher’s exact test**
HH elevation (m)^a^	1854 (1821–1907)	1846 (1818–1893)	1851 (1820–1904)	0.426
1701–1900	224, 73.9 (68.7–78.6)	230, 75.9 (70.7–80.4)	454, 74.9 (71.3–78.2)	
1901–2100	72, 23.8 (19.3–28.9)	70, 23.1 (18.7–28.2)	142, 23.4 (20.2–27.0)	
> 2100	7, 2.3 (1.1–4.8)	3, 1.0 (0.3–3.0)	10, 1.7 (0.9–3.0)	
House proximity to Lake Tana (km)^a^	6.5 (5.4–9.5)	6.9 (5.3–8.8)	6.8 (5.3–8.9)	0.720
< 2	18, 5.9 (3.8–9.3)	23, 7.6 (5.1–11.2)	41, 6.8 (5.0–9.1)	
2–4	22, 7.3 (4.8–10.8)	22, 7.3 (4.8–10.8)	44, 7.2 (5.4–9.6)	
> 4	263, 86.8 (82.5–90.2)	258, 85.1 (80.7–88.7)	521, 86.0 (83.0–88.5)	
House proximity to water body 1 (m)^a^	823 (548–1372)	918 (530–1482)	858.5 (545–1417)	0.345
< 250	18, 5.9 (3.8–9.3)	13, 4.3 (2.5–7.3)	31, 5.1 (3.6–7.2)	
250–500	43, 14.2 (10.7–18.6)	54, 17.8 (13.9–22.6)	97, 16.0 (13.3–19.2)	
> 500	242, 79.9 (74.9–84.0)	236, 77.9 (72.8–82.2)	478, 78.9 (75.4–82.0)	
House proximity to water body 2 (m)^a^	1770 (909–2343)	1783 (967–2432)	1776.5 (932–2383)	0.634
< 250	10, 3.3 (1.8–6.0)	12, 4.0 (2.3–6.9)	22, 3.6 (2.4–5.5)	
250–500	20, 6.6 (4.3–10.0)	15, 5.0 (3.0–8.1)	35, 5.8 (4.2–7.9)	
> 500	273, 90.1 (86.2–93.0)	276, 91.0 (87.3–93.8)	549, 90.6 (88.0–92.7)	

**p < 0.05 was considered statistically significant

^a^Continuous variables; summarized through median and IQR

### Mantel–Haenszel analysis for proving co-factors and interaction

The stratified analysis confirmed that there was no significant variation between stratum-specific ORs, indicating no interaction between the predictors and HH elevation or travel to malarious lowlands. Stratified analysis also revealed no disparity between the pooled and adjusted ORs, indicating that HH elevation was not a co-factor for the effect of predictors on malaria. Nonetheless, travel to malarious lowlands was a co-factor for the effects of age, marital status, occupation, educational status, number of rooms, travel history within the study area, HH member’s travel to malarious lowlands, inappropriate HMM, IRS, and HH elevation on malaria (Tables 5 and 6). Additional table files show the determinants of malaria by HH elevation and travel to malarious lowlands (see Additional files 2 and 3).

**Table 5:** Stratified analysis by HH elevation for predictors of malaria in villages around Lake Tana, Ethiopia, October 2016–June 2017

Variables	COR	[1750–2000 m]	[2000–2500 m]	Adjusted (Mantel–Haenszel)			
		OR	OR	OR_MH_	95% CI	X^2^_MH_	*p* value
Age	1	0.97	2.5	1.01	0.73–1.40	0.97	0.3237
Marital status	1.62	1.69	1.5	1.68	1.10–2.56	0.02	0.8792
Occupation	1.19	1.22	0.79	1.19	0.77–1.84	0.17	0.6762
Educational status	1.34	1.31	5.45	1.36	0.98–1.88	1.5	0.2205
HH size	1.1	1.08	1.75	1.1	0.80–1.52	0.35	0.5523
HH wealth index	1.29	1.29	1.24	1.29	0.91–1.82	0	0.9629
Roofing materials	0.62	0.6	1.25	0.62	0.32–1.19	0.24	0.6233
Open eaves in the roof	0.86	0.79	3.11	0.87	0.54–1.40	1.96	0.1615
Number of rooms	1.11	1.17	0.3	1.09	0.78–1.52	2.65	0.1035
Separate kitchen	1.09	1.13	0.7	1.1	0.79–1.53	0.36	0.5464
Tethering of cattle, goats and sheep	1.08	1.09	0.98	1.08	0.74–1.58	0.02	0.8995
Travel history to malarious lowlands	2.81	3.3	0.98	2.91	1.73–4.90	2.14	0.1436
Travel history within the study area	1.47	1.52	1.17	1.49	1.04–2.15	0.11	0.737
Visiting traditional healers	0.84	0.88	0.57	0.85	0.57–1.28	0.27	0.6019
HMM	1.14	1.15	1.38	1.17	0.70–1.94	0.05	0.8304
Received health education on malaria	1.54	1.55	1.43	1.55	1.10–2.17	0.01	0.9153
LLIN ownership	1.26	1.28	0.9	1.26	0.89–1.78	0.15	0.6984
IRS	0.98	0.95	0	0.94	0.63–1.39	0.77	0.3805
House proximity to health centre	0.71	0.7	1.11	0.71	0.49–1.04	0.26	0.6089
House proximity to water body 1	0.71	0.79	0.32	0.68	0.33–1.43	0.7	0.4024
House proximity to water body 2	1.21	1.13	1.71	1.19	0.50–2.81	0.09	0.7623

*OR*_*MH*_ adjusted odds ratio using Mantel–Haenszel method, *COR* crude odds ratio, *X*^*2*^_*MH*_ Mantel–Haenszel Chi square value

**Table 6:** Stratified analysis by travel to malarious lowlands for predictors of malaria in villages around Lake Tana, Ethiopia, October 2016–June 2017

Variables	COR	Travel to malarious lowlands		Adjusted (Mantel–Haenszel)			
		Travel history (OR)	No travel history (OR)	OR_MH_	95% CI	X^2^_MH_	*p* value
Age	1	0	0.82	0.8	0.57–1.13	1.02	0.3123
Marital status	1.62	0.93	1.45	1.32	0.86–2.04	0.66	0.4182
Occupation	1.19	1.34	0.92	0.99	0.63–1.57	0.41	0.5242
Educational status	1.34	1.24	1.21	1.21	0.88–1.68	0	0.9725
HH size	1.1	0.99	1.18	1.16	0.84–1.60	0.11	0.7423
HH wealth index	1.29	1.21	1.26	1.26	0.89–1.78	0.01	0.9375
Roofing materials	0.62	0.38	0.66	0.61	0.32–1.17	0.36	0.547
Open eaves in the roof	0.86	1.02	0.79	0.82	0.50–1.33	0.13	0.7152
Wall building materials	0.71	0	1.09	0.77	0.22–2.73	1.23	0.2669
Flooring materials	0.44	0	0.58	0.45	0.13–1.60	0.71	0.401
Number of rooms	1.11	1.14	1.26	1.25	0.88–1.76	0.04	0.8482
Separate kitchen	1.09	0.9	1.04	1.02	0.73–1.43	0.08	0.7838
Tethering of cattle, goats and sheep	1.08	0.74	1.09	1.03	0.71–1.51	0.45	0.5
Travel history within the study area	1.47	0.29	1.18	1.03	0.68–1.56	3.08	0.0792
HH member travel to malarious lowlands	3.46	2.6	2.56	2.58	1.16–5.74	0	0.9841
Visiting traditional healers	0.84	0.93	0.79	0.8	0.53–1.22	0.08	0.7788
HMM	1.14	0.9	0.92	0.91	0.54–1.54	0	0.9821
Received health education on malaria	1.54	2.43	1.31	1.42	1.00–1.99	1.32	0.2508
LLIN ownership	1.26	1.07	1.16	1.15	0.80–1.64	0.02	0.8749
IRS	0.98	2.19	1	1.13	0.78–1.65	2.12	0.1453
HH elevation	0.8	0.28	0.93	0.67	0.32–1.40	2.05	0.1526
House proximity to health centre	0.71	0.56	0.75	0.73	0.50–1.05	0.25	0.6138
House proximity to Lake Tana	1.3	1.54	1.37	1.4	0.73–2.69	0.02	0.8918
House proximity to water body 1	0.71	0.98	0.73	0.77	0.37–1.62	0.09	0.7599
House proximity to water body 2	1.21	1.7	1.26	1.34	0.56–3.19	0.08	0.7812

*OR*_*MH*_ adjusted odds ratio using Mantel–Haenszel method, *COR* crude odds ratio, *X*^*2*^_*MH*_ Mantel–Haenszel Chi square value

### Multivariable modelling through conditional logistic regression

Multivariable adjustment established that travel to malarious lowlands in the preceding month (adjusted mOR = 7.32; 95% CI 2.40–22.34), HH member travel to malarious lowlands (adjusted mOR = 2.75; 95% CI 1.02–7.44), and delivery of health information on malaria (adjusted mOR = 1.57; 95% CI 1.03–2.41) were independently associated with malaria heterogeneity (Table 7).

**Table 7:** Determinants of malaria transmission dynamics in villages around Lake Tana, Ethiopia, October 2016–June 2017

Variables	*β*	Unadjusted mOR^a^	*p*-value	*β*	Adjusted mOR (95% CI)^b^	*p*-value
Age (years)	− 0.02	0.98	0.125	− 0.02	0.98 (0.95–1.01)	0.183
Marital status						
Married		1			1	
Single	0.52	1.68	*0.057*	0.31	1.37 (0.65–2.87)	0.410
Divorced/widowed	0.68	1.97	0.101	0.64	1.89 (0.71–5.02)	0.200
Occupation						
Farmer		1			1	
Non-farmer	− 0.20	1.23	0.400	− 0.23	0.79 (0.39–1.60)	0.513
Educational status						
Illiterate		1			1	
Able to read and write (informal)	0.32	1.38	0.163	0.38	1.46 (0.87–2.44)	0.151
Grade 1 to 12 completed	0.43	1.54	*0.042*	0.04	1.04 (0.62–1.73)	0.885
>12	− 0.33	0.72	0.535	− 0.53	0.59 (0.15–2.30)	0.447
HH wealth index						
Poor		1			1	
Medium	0.48	1.62	0.197	0.36	1.43 (0.62–3.27)	0.400
Rich	0.26	1.29	0.162	0.21	1.24 (0.82–1.88)	0.316
Roofing materials						
Corrugated iron		1			1	
Thatch	− 0.56	0.57	0.122	− 0.31	0.73 (0.32–1.68)	0.459
Flooring materials						
Cement and others		1			1	
Dung plastered	− 0.81	0.44	0.177	− 0.83	0.42 (0.11–1.78)	0.248
Travel to malarious lowlands						
No		1			1	
Yes	2.28	9.75	*< 0.0001*	1.99	7.32 (2.40–22.34)	< 0.0001
Travel history within the study area						
No		1			1	
Yes	0.45	1.56	*0.026*	− 0.03	0.97 (0.59–1.58)	0.900
HH member travel to malarious lowlands						
No		1			1	
Yes	1.35	3.86	*0.001*	1.01	2.75 (1.02–7.44)	0.046
Received health information on malaria						
No		1			1	
Yes	0.46	1.59	*0.010*	0.45	1.57 (1.03–2.41)	0.037
LLIN(s) ownership						
None		1			1	
1 LLIN/household	0.07	1.07	0.674	0.11	1.12 (0.66–1.90)	0.684
≥ 2 LLINs/household	0.63	1.87	*0.021*	0.59	1.81 (0.95–3.46)	0.071
HH elevation (m)						
1701–1900		1			1	
1901–2100	0.34	1.40	0.355	− 0.09	0.91 (0.36–2.31)	0.844
> 2100	1.12	3.06	0.137	0.66	1.94 (0.30–12.49)	0.484
House proximity to health centre (km)						
≤ 5		1			1	
> 5	− 0.34	0.71	0.069	− 0.27	0.76 (0.49–1.18)	0.226
House proximity to water body 1 (m)						
< 250		1			1	
250–500	− 0.52	0.59	0.204	− 0.18	0.84 (0.30–2.32)	0.734
> 500	− 0.28	0.75	0.459	− 0.03	0.97 (0.38–2.47)	0.950
House proximity to water body 2 (m)						
< 250		1			1	
250–500	0.44	1.55	0.409	0.59	1.81 (0.50–6.57)	0.365
> 500	0.14	1.15	0.740	0.39	1.48 (0.51–4.32)	0.475
House proximity to Lake Tana (km)						
< 2		1			1	
2–4	0.28	1.32	0.552	0.28	1.33 (0.46–3.81)	0.596
> 4	0.29	1.33	0.399	0.37	1.45 (0.66–3.16)	0.353

*β*: beta coefficients/regression coefficients. *p *< 0.05 was considered statistically significant. The italics indicate statistically significant *p*-values

Unadjusted mOR: Unadjusted matched odds ratio; adjusted mOR: adjusted matched odds ratio; 1: Reference category

^a^Denotes unadjusted mOR using 95% CI in bivariate conditional logistic regression analysis from matched case–control pairs

^b^Denotes adjusted mOR using 95% CI in multivariable conditional logistic regression analysis from matched case–control pairs

Age of patients; marital status, occupation, and educational status of the patient/HH head; HH size; HH wealth index; flooring, roofing, and wall building materials; number of rooms; separate kitchen; window types; open eaves in the roof; tethering of cattle, goats, and sheep at night; travel history within the study area; visits to traditional healers; inappropriate HMM; LLIN ownership; IRS; HH elevation; house proximity to health centre; house proximity to water body 1; house proximity to water body 2; and house proximity to Lake Tana were not significantly associated with malaria infection (*p *> 0.05). In other words, there were no significant differences between cases and controls with respect to these characteristics (Table 7).

## Discussion

In this study, travel history to malarious lowlands in the preceding month, having a HH member who travelled to malarious lowlands in the preceding month, and delivery of health information on malaria were statistically significant and independent predictors of malaria in villages near Lake Tana, northwest Ethiopia.

Individuals who travelled to malarious areas had higher odds of malaria infection than those who did not travel. This finding agrees with that of other studies in Ethiopia [7, 14] and in Zanzibar [47]. This may be due to the susceptibility of travellers from the midlands to malaria that progresses to severe symptomatic malaria, especially among those travellers who slept without personal protective measures. Infection-naïve individuals are highly susceptible to malaria if exposed to an infectious anopheles mosquito, particularly in hyper-endemic areas [48]. The travels mainly take place during high malaria transmission periods among people seeking temporal job and engaging in social activities. This and other studies showed low chemoprophylaxis and LLIN use during travels [7]. In the hot lowlands, many labourers sleep outdoors and in open spaces without protection from mosquitoes, resulting in high malaria exposure risk. Large numbers of imported malaria cases have been found in the northwestern Ethiopian highlands [7]. Thus, imported malaria is a health risk besides locally acquired malaria in midland villages around Lake Tana. This duality of malaria origins is also indicated by the fact that three-quarters of the malaria cases could not be traced to the lowlands. This epidemiological situation points to a critical obstacle to eliminating malaria through targeted interventions.

Individuals who did not know malaria aetiology, transmission, and prevention methods had an increased odds of risk for malaria infection. The possible explanation for this finding may be the low educational status of the study population or delivery of malaria information independent of educational status, which prevents them from understanding and analysing the information provided to them by community health workers.

In the multivariable adjustment, sociodemographic factors, HH wealth, housing structure, travel history within the study area, visits to traditional healers, inappropriate HMM, LLIN ownership, IRS, HH elevation, and house proximity to water bodies were not associated with malaria. This might be due to low heterogeneity of housing structure, socio-demographic status, HH wealth, proximity to water bodies, elevation of HHs, and delivery of inadequate information on treatment-seeking, bed net use, and other preventive measures by Gondar Zuriya District Health Office. Demographically, educational status did not vary significantly between cases and controls. This corroborates the findings of other studies in Ethiopia [49] and in Gambia [9], although advancement in education confers significant protection from malaria [50]. Occupation was not a significant predictor of malaria infection. This may have been due to the small number of non-agricultural workers in the study. However, another study in the Ethiopian highlands showed that agricultural workers were at elevated risk of malaria [7]. The difference in findings may be due to sample size. Interestingly, marital status was marginally significantly associated with malaria infection in bivariate analysis. This may be because mostly unmarried males travel to the malarious lowlands for work. In addition, unmarried people are generally in poorer health and have a higher mortality risk than married people [11].

In this study, HH size played no significant role in the odds of contracting malaria. This finding is consistent with that of other studies in Ethiopia [51] and Malawi [8]. Several studies associated larger HH size with malaria [52]. HH wealth facilitates the management of illness, and several studies reported better access to health care by wealthier groups [12, 51]. Nonetheless, HH wealth had no significant impact on malaria in this study.

Housing structure had no significant statistical association with malaria transmission, a finding also reported in previous studies [8, 9]. However, researchers have reported that individuals living in houses with mud walls [13], thatched roofs [53], dung plastered floors [53], and boarded windows had higher risk of malaria [9]. Surprisingly, spatial malaria heterogeneity was not determined by fine-scale environmental factors, particularly water bodies close to HHs. This study is in agreement with a study that stated malaria incidence was not associated with distance to an Ethiopian dam reservoir [22]. Two other studies in Ethiopia reported that the odds of malaria infection were lower in HHs closer to reservoirs than those at greater distances [20, 21]. On the other hand, numerous studies have found large numbers of malaria cases in HHs near various types of water bodies [16, 18, 19]. Possible reasons for these contradictory findings may be differences in the use of LLINs or IRS, socioeconomic and environmental factors in populations, vulnerabilities or transmission dynamics, differential health services accessibility, or imported malaria cases.

In this study, LLIN ownership and IRS had no significant effect on malaria prevalence. This finding is similar to that of studies revealing that LLIN ownership and use as well as indoor wall spraying did not protect against malaria [7, 9, 20, 25]. However, other studies reported a protective effect of bed nets and IRS on malaria [8, 15, 25, 53]. The difference may be due to failure to use LLINs properly and consistently, inadequacy or poor condition of LLINs, or failure to use them during travel. As a result, the protective effect of LLINs could have been negated [33]. Besides, imported malaria, human activities at night outside the home, the spread of insecticide-resistant vectors, and spraying by untrained individuals and substandard insecticides may have reduced the protective effect of IRS.

A matched case–control study was used to eliminate the confounding effects of sex and age, which solidified the conclusions and recommendations. Matched case–control studies have minimal bias relative to cross-sectional and unmatched case–control designs because they compare groups internally. The water bodies proximate to the HHs were georeferenced through direct ecological surveys to increase measurement accuracy despite accessible satellite imagery. To avoid information bias, data collectors were blinded to the malaria status of patients during onsite data collection at the HH level. All other measurements of exposure variables were performed at home through interviews and direct observations. Stratified analyses and multivariable conditional logistic regression were used to control confounding and report interaction effects.

This study has several limitations. It is difficult to establish temporal relationships between exposures and malaria. Employing PCD to measure the effect of house proximity to water bodies on spatial malaria heterogeneity was not efficient due to the presence of asymptomatic and submicroscopic parasite carriers and the treatment of malaria by drug vendors, private clinics, and health posts [50], which were not studied. The asymptomatic parasite carriage may influence the risk factor analysis, especially if the reservoirs occur more often in controls than in cases. This study failed to obtain information on the size and nature of water bodies. Case and control selection from health institutions can be prone to bias since patients usually do not represent the general population. Recall bias could have been introduced during travel history measurement. The unmeasured residuals were not controlled but the effect on the finding is marginal. Moreover, due to unrest in the study area, the study did not include the month of September, at the end of the main rainy season, when more imported and locally acquired malaria cases occur in northwestern Ethiopia [54]. Even though this is a low transmission setting, travel history associated with malaria does not necessarily identify imported cases.

These study findings emphasize the fact that comprehending the sociodemographic characteristics of individuals who travel to malarious lowlands, travel patterns in individual villages, adequacy of health information on malaria, and taking into account the potential vector breeding sites in mid-altitude villages are central to facilitating malaria elimination strategies.

## Conclusions

Seasonal and other short-term migration to commercial agricultural farms in the malarious Ethiopian lowlands is a significant independent risk factor for malaria infection in villages around Lake Tana. In addition, inadequate health information on malaria and marital status, apparently related to the predominance of unmarried young males migrating to the lowlands, are risk factors for malaria infection. There is a need for studies to explore risk factors of imported malaria and locally acquired malaria independently in the midlands of northwestern Ethiopia. In addition, information is needed on the transmission dynamics of imported malaria in the midlands. This information is critical for designing effective and integrated interventions that consider imported malaria cases and approaches to accessing health information on malaria control in northwest Ethiopia and other highland/lowland transitional areas.

## Additional files

**Additional file 1.** Study *kebeles* characteristics. a) A section of the study area bordering Lake Tana. b) A stagnant water potential for *Anopheles* mosquitoes breeding. c) Use of LLINs for protection of straw. d) An LLIN they showed packed in its plastic bag.

**Additional file 2.** Characteristics of determinants of malaria by HH elevation in villages around Lake Tana, Ethiopia, October 2016-June 2017.

**Additional file 3.** Characteristics of determinants of malaria by travel to lowlands in villages around Lake Tana, Ethiopia, October 2016-June 2017.

## Abbreviations

ACT: artemisinin-based combination therapy
AOR: adjusted odds ratio
COR: crude odds ratio
HH: household
HMM: home based management malaria
IQR: interquartile range
IRS: indoor residual spraying
LLINs: long-lasting insecticidal nets
MDG: Millennium Development Goals
mOR: matched odds ratio
PCA: principal component analysis
PCD: passive case detection
RDTs: rapid diagnostic tests
WHO: World Health Organization
