# Supplementary material for: Travel to farms in the lowlands and inadequate malaria information significantly predict malaria in villages around Lake Tana, northwest Ethiopia: a matched case–control study
Source: Malar J. 2018 Aug 10;17:290. doi: 10.1186/s12936-018-2434-y (PMC6086053; doi:10.1186/s12936-018-2434-y)
Supplement: Supplementary file 1 — Additional file 1. Study kebeles characteristics. a) A section of the study area bordering Lake Tana. b) A stagnant water potential for Anopheles mosquitoes breeding. c) Use of LLINs for protection of straw. d) An LLIN they showed packed in its plastic bag. [file 12936_2018_2434_MOESM1_ESM.pdf]

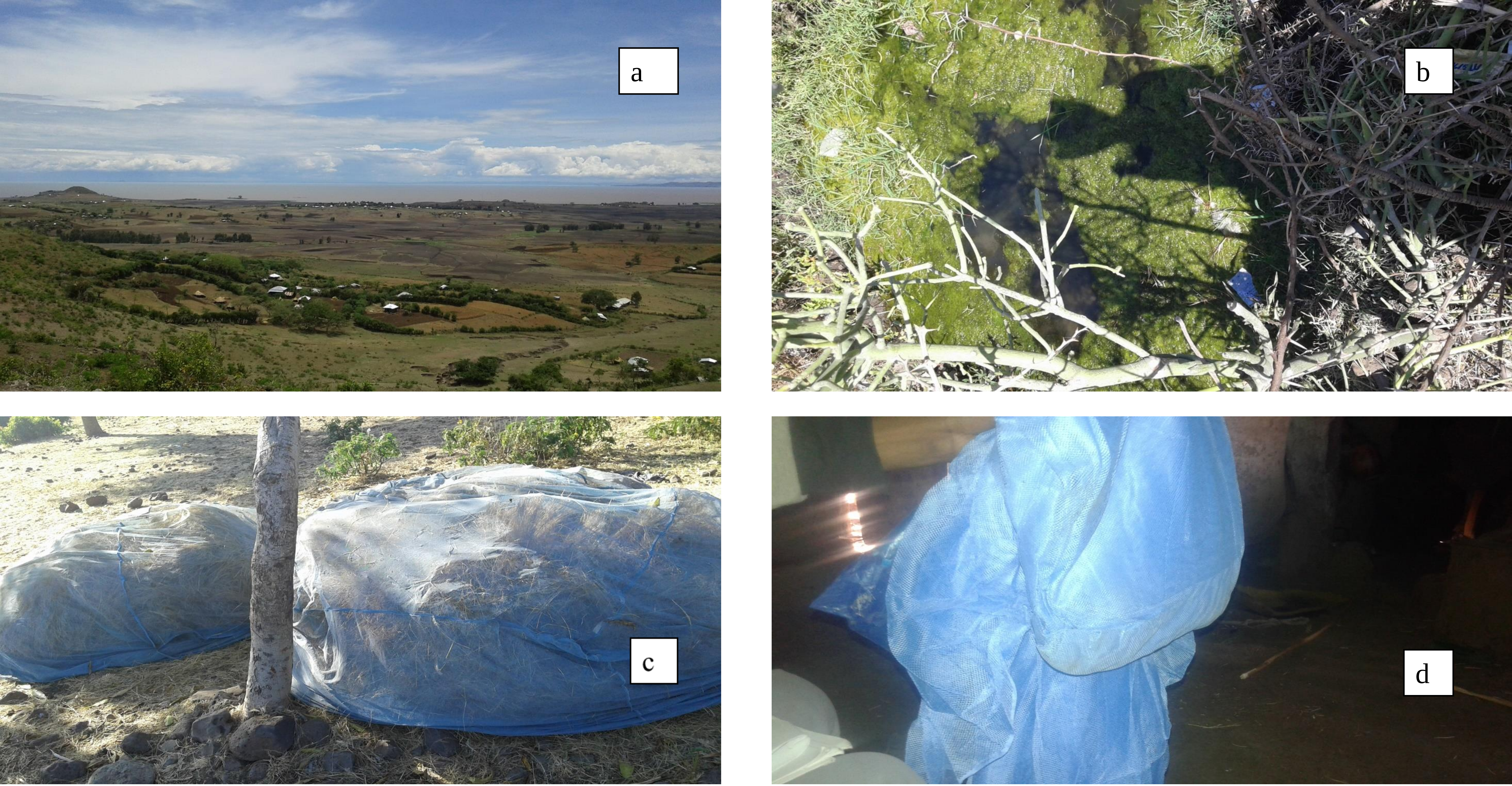

Figure S1. Study *kebeles* characteristics: a) A section of the study area bordering Lake Tana. b) A stagnant water potential for *Anopheles* mosquitoes breeding. c) Use of LLINs for protection of straw. d) An LLIN they showed packed in its plastic bag.
